# The Scavenger Function of LSECs, a Regulator of Liver Diseases

**DOI:** 10.3390/cimb48080802

**Published:** 2026-08-07

**Authors:** Juntao Zhou, Lijuan Zhang, Jianqiao Wang, Chengliang Zhang, Cheng Tian

**Affiliations:** 1 Department of Pharmacy, Tongji Hospital, Tongji Medical College, Huazhong University of Science and Technology, Wuhan 430030, China; zjt20250108@126.com (J.Z.); joanne1016213@163.com (J.W.); 2 School of Pharmacy, Xianning Medical College, Hubei University of Science and Technology, Xianning 437100, China; 14753065273@163.com

**Keywords:** LSECs, MASLD, ALD, liver fibrosis, HCC, scavenger receptors, therapeutic strategies

## Abstract

Liver sinusoidal endothelial cells are highly specialized endothelial cells that contribute to liver homeostasis through their remarkable scavenger function. Through a broad repertoire of scavenger receptors, including mannose receptor, scavenger receptor H, fragment crystallizable gamma receptor IIb, LSECs efficiently clear waste and toxins, altering drug distribution and metabolism. However, this scavenger function can be disrupted by aging, toxins, and transcription factors, thereby promoting the accumulation of harmful substances within the hepatic microenvironment. Impaired LSEC scavenger function contributes to the initiation and progression of multiple liver diseases, such as metabolic dysfunction associated steatotic liver disease, alcoholic liver disease, liver fibrosis, hepatocellular carcinoma, and antibody-based drug-related liver injury. Emerging pharmacological evidence suggests that antioxidant agents, anti-angiogenic strategies and anti-inflammatory interventions may partially restore the expression or function of LSEC scavenger receptors to alleviate liver diseases. In summary, the scavenger function of LSECs serves as a key gatekeeper in liver homeostasis, and targeted modulation of this function holds great potential for the treatment of liver diseases.

## 1. Introduction

Liver sinusoidal endothelial cells (LSECs) are specialized endothelial cells characterized by the absence of a continuous basement membrane and intercellular septa, with small pore structures called fenestrae on their cell membranes [1]. Despite comprising only approximately 3.3% of the total liver cell volume, LSECs represent 55% of non-parenchymal liver cells and carry out indispensable functions in substance exchange and clearance, immune modulation, hemodynamic regulation, hepatocyte proliferation and maintenance of hepatic stellate cells (HSCs) quiescence within the liver [2,3]. Notably, recent studies suggest that LSECs, with their unique scavenging function, are critically involved in various liver conditions [4,5,6].

The liver serves as a primary organ responsible for metabolism and detoxification, orchestrating the metabolism of lipids, proteins and carbohydrates, as well as the clearance of drugs and toxins from the circulation [7]. However, during the process of metabolism, the accumulation of harmful substances can cause liver injury. For instance, dysregulated lipid metabolism promotes lipotoxic accumulation and oxidative injury, contributing to the development of metabolic dysfunction associated steatotic liver disease (MASLD) [8]. Similarly, excessive alcohol intake generates acetaldehyde and reactive oxygen species (ROS), leading to the onset of alcoholic liver disease (ALD) [9]. Both of these liver conditions can progress to hepatitis, liver fibrosis, cirrhosis, and even hepatocellular carcinoma (HCC) [10,11]. In addition, during the process of drug metabolism and detoxification, various medications and their reactive metabolites can precipitate drug-induced liver injury (DILI) [12]. Importantly, LSECs are key regulators of the pathogenesis of these conditions, as the impairment of their scavenger function undermines hepatic homeostasis and accelerates the progression of liver diseases [4].

The hepatic reticuloendothelial system is generally considered to include two specialized clearance cell populations, Kupffer cells (KCs) and LSECs [13]. KCs are liver-resident macrophages endowed with potent phagocytic capacity that enables them to clear pathogens such as bacteria and viruses, damaged cells or their components, and diverse particles through phagocytosis [14,15]. Compared to KCs, LSECs are particularly adept at scavenging small molecules less than 200 nm in size, thereby complementing the phagocytic function of KCs [16]. Although an individual LSEC exhibits lower clearance efficiency than a single KC, the numerical predominance and expansive luminal surface area of LSECs render their aggregate contribution to hepatic clearance comparable to that of KCs, enabling them to play an important role in detoxification, metabolic balance and immune regulation [17].

Given the critical and unique physiological role of LSEC scavenger function, previous reviews have provided important foundations for understanding LSEC scavenger function. Pandey et al. catalogued the principal scavenger receptors expressed by LSECs and discussed their structural features, ligands, physiological functions, and alterations in liver homeostasis and disease [18]. Bhandari et al. subsequently focused on the blood-clearance function of LSECs, particularly the uptake of circulating waste macromolecules and nanoparticles, species-related differences, and the unintended clearance of nanomedicines and biologics [3]. However, these reviews did not summarize the mechanisms by which LSEC scavenger dysfunction promotes liver diseases and the potential therapeutic agents that might alleviate liver diseases by improving LSEC scavenger dysfunction. In this review, we focus on the mechanism of liver diseases induced by LSEC scavenger dysfunction, and the potential therapeutic agents targeting this dysfunction. Overall, this review provides an updated overview of the classification of relevant scavenger receptors, the physiological roles of LSEC-mediated scavenging, factors contributing to dysregulated scavenger function in LSECs, the consequences of such dysfunction in liver diseases, and pharmacological strategies for restoring LSEC scavenger function.

## 2. LSEC Scavenger Function

### 2.1. Classification and Function of LSEC Scavenger Receptors

**Figure 1:**
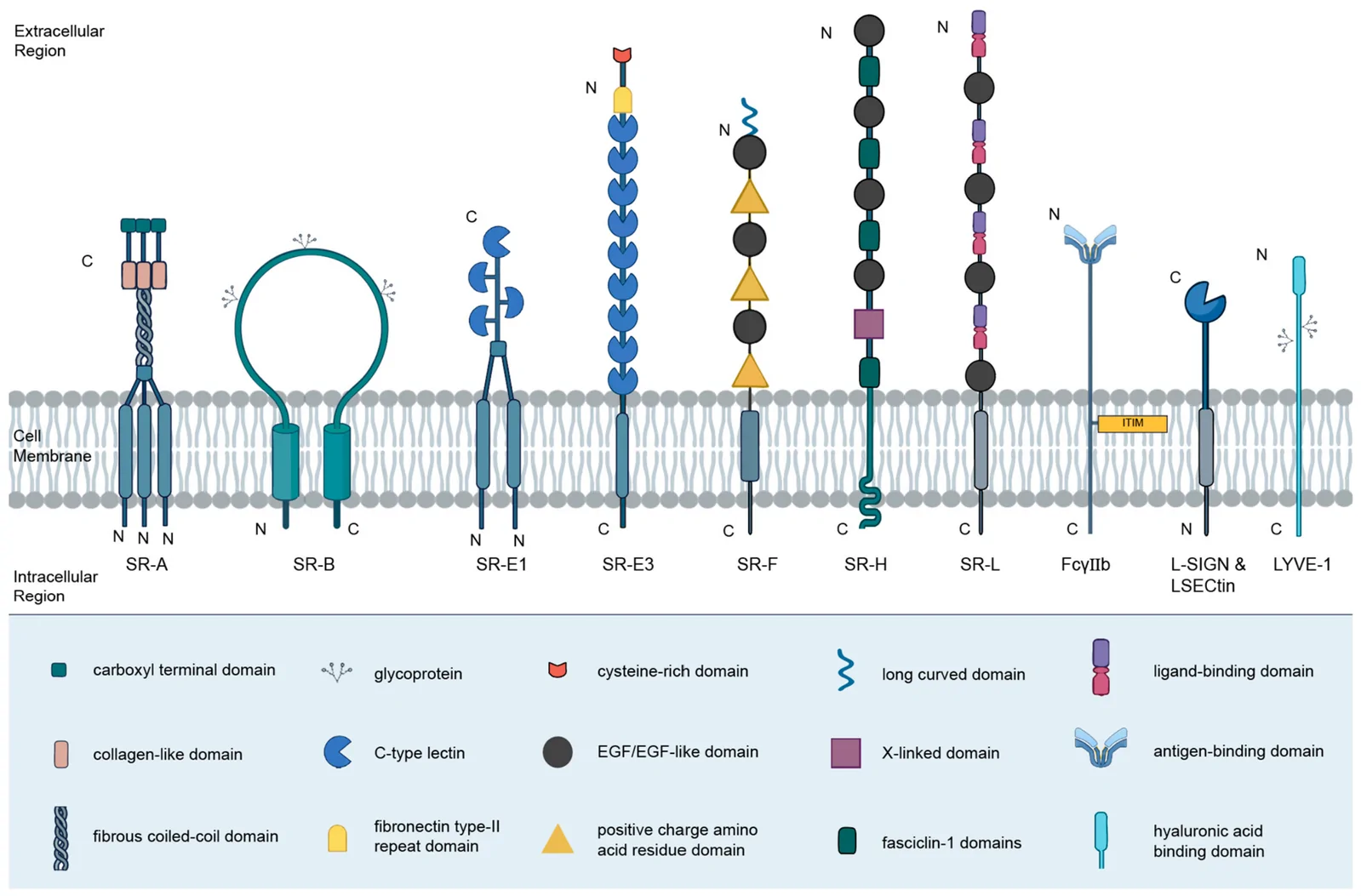
Classification and structural features of scavenger receptors in LSECs. (**1**) SR-A family is a type II trimeric integral membrane receptor, with a carboxyl terminal domain, collagen-like domain, and fibrous coiled-coil domain related to its endocytic function. (**2**) SR-B family is a membrane glycoprotein composed of two short cytoplasmic N-terminal and C-terminal domains, two short hydrophobic membrane regions, and a highly N-glycosylated large circular extracellular domain. (**3**) SR-E1 is a type II transmembrane protein that exists in dimeric form, and its recognized ligand is related to the C-type lectin structural domain. (**4**) The mannose receptor is a type I transmembrane protein, and its recognized ligands are primarily determined by C-type carbohydrate recognition domains, fibronectin type II repeat domain, and cysteine-rich domain. (**5**) SR-F family is a type I transmembrane protein, and the ligands it can recognize are mainly determined by the EGF/EGF-like domain and the positive charge amino acid residue domain (**6**) SR-H family is a type I transmembrane protein, and its recognizable ligands are mainly determined by the EGF/EGF-like domain and fasciclin-1 domains. (**7**) SR-L is a type I transmembrane protein, and its recognizable ligands are mainly determined by the EGF/EGF-like domain and the ligand-binding domain. (**8**) FcγRIIb is a type I transmembrane protein with an IgG recognition site and an immunoreceptor tyrosine-based inhibitory motif (ITIM). (**9**) LSECtin and L-SIGN are type II transmembrane receptors, and the type of ligand they can recognize is determined by the C-type carbohydrate recognition domain. (**10**) LYVE-1 is a type I integral membrane glycoprotein containing a single link module in its extracellular domain responsible for binding HA.

LSEC scavenging is mediated largely by clathrin-dependent receptor endocytosis [3,13]. The receptor repertoire includes the classical scavenger receptor families scavenger receptor A (SR-A), SR-B, SR-E, SR-F, SR-H and SR-L, together with the complementary clearance receptors Fc gamma receptor IIb (FcγRIIb), liver sinusoidal endothelial cell lectin (LSECtin), liver/lymph node-specific intercellular adhesion molecule-3 grabbing non-integrin (L-SIGN) and lymphatic vessel endothelial hyaluronan complexes receptor 1 (LYVE-1). These proteins are divided into type 1 transmembrane proteins and type 2 transmembrane proteins. Their key structural features, representative ligands and principal functions are summarized in Table 1. The main topological features of each receptor are shown in Figure 1. This structural diversity enables LSECs to recognize modified lipoproteins, glycoconjugates, extracellular-matrix products, immune complexes and antigens on the surface of bacteria and viruses, which plays an important role in metabolism, detoxification and immune regulation.

### 2.2. The Physiological Role of LSEC Scavenger Function

#### 2.2.1. Physiological Metabolism Regulation

LSECs possess unique structural characteristics that confer high permeability and facilitate the efficient clearance of waste products from the blood through high-affinity scavenger receptors [2,4]. Each type of receptor demonstrates a certain preference for specific ligands due to its unique structure. This diversity of receptors enables LSECs to recognize and internalize a wide range of ligands, which is typically accomplished through the dynamin-dependent invagination of clathrin-coated pits [50].

It is indicated that LSECs maintain the metabolic balance of carbohydrates, lipids and proteins [19]. These substances are degraded by lysosomes after being endocytosed, thereby preventing the accumulation of potentially harmful materials in the liver and circulatory system [50,51,52]. Specifically, scavenger receptors like MR and SR-H2 mediate the clearance of carbohydrates, with the clearance of glycosaminoglycans such as HA, chondroitin sulfate, and heparin primarily mediated by SR-H2 [53,54]. Multiple scavenger receptors are involved in the internalization of proteins. Respectively, SR-A, MR, and SR-H2 mediate the clearance of β-amyloid fibrils, FSA, and HOA, while SR-B2 is responsible for the clearance of collagen [27,37,55,56]. Notably, research indicates that LSECs rely on MR to take up lysosomal enzymes from the bloodstream in order to maintain their high degradative capacity [27]. Furthermore, LSECs not only directly take up lipid-soluble vitamins but also regulate lipid metabolic balance by modulating lipoprotein clearance, with SR-B, SR-H, and LOX-1 playing principal roles in this process [36,57]. These receptors clear lipids from the circulation by regulating the levels of VLDL, LDL and modified LDL in plasma [58,59,60]. In addition, the SR-B family in LSECs also participates in the regulation of plasma HDL [61].

#### 2.2.2. Drug and Toxin Metabolism

It is currently understood that hepatocytes are the primary cells responsible for drug and toxin metabolism [62]. However, LSECs also play a partial role in drug metabolism. Besides their scavenger receptors, LSECs express cytochrome P450 family 1 subfamily B member 1 (CYP1B1) and CYP2E1 [63,64]. On this basis, studies find that they can metabolize acetaminophen and alcohol prior to hepatocytes, thereby reducing the toxic effects on hepatocytes [65].

In addition, LSECs also influence the distribution and metabolism of anionic nanodrugs and antibody-based drugs. Through SR-H2, LSECs can recognize the doxorubicin encapsulated in myocet and the amphotericin B encapsulated in liposome, facilitating endocytosis and affecting the distribution and subsequent metabolic processes of these drugs [66]. Studies have also shown that when high doses of ligands are administered, the binding sites of scavenger receptors on LSECs become saturated [55]. This saturation can also exert a competitive inhibitory effect on the binding of other ligands [55]. For instance, racemic thiosugar sulfate inhibits the uptake of anionic nanoparticles by SR-H2 as a competitive inhibitor, which has profound implications for the rational design of nanocarriers [66]. Moreover, evidence suggests that LSECs modulate the clearance of therapeutic antibodies such as immune checkpoint inhibitors through FcγRIIb [39]. This interaction alters pharmacokinetic profiles and can ultimately attenuate therapeutic efficacy [67].

#### 2.2.3. Immune Regulation

LSECs possess a powerful capacity for material clearance and can directly eliminate blood-borne pathogenic microorganisms such as bacteria, viruses and fungi, along with their pathogenic components [52]. They can recognize glycoproteins present on the surface of pathogens, mediating the endocytosis of various agents including bacteriophages, adenoviruses, HCV, HIV, SARS-CoV, Ebola virus, *Candida albicans* and *Escherichia coli* [17,52,68,69]. When pathogen exposure is limited, this process quickly eliminates them with a typically silent effect, thus preventing unnecessary immune activation [40]. In addition, LSECs can also clear antibody-pathogen immune complexes through FcγRIIb to maintain immune homeostasis [70,71,72].

Beyond their powerful antigen-clearing capability, LSECs also function as potent unconventional antigen-presenting cells through scavenger receptors [73]. LSECs can sequester antigens with an efficiency up to 100-fold higher than that of dendritic cells, regulating immune responses in a manner that often promotes immune tolerance rather than activation, particularly for circulating soluble antigens like lipopolysaccharides (LPS) [74]. It is indicated that SR-H1 mediates the clearance of LPS, suppresses immune responses and limits toll-like receptor 4 (TLR4)-mediated inflammation [34]. In addition, LSECtin in LSECs significantly decreases the phosphorylation levels of key tyrosine kinases during T cell receptor activation, thereby inhibiting T cell activation and proliferation, and consequently alleviating inflammatory damage to the liver [75]. The MR in LSECs facilitates the clearance of antigens like LPS from the bloodstream, promoting the uptake and presentation of these antigens [76]. Ultimately, by engaging in cross-presentation of antigens, LSECs can induce immune tolerance in CD8^+^ T cells [76]. This function suppresses inflammatory responses and also promotes the immune evasion of tumor cells [77]. Notably, while promoting tolerance, LSECs have the capacity to transform CD8^+^ T cells into unique memory cells, which are not only spared from clearance by immature dendritic cells, but also exhibit characteristics similar to those of central memory T cells [78]. This process can occur even in the absence of systemic inflammation, ensuring an effective immune response during infections, thus providing a balance between tolerance and immunity [77,78].

## 3. Factors Contributing to Dysregulated LSEC Scavenger Function

The scavenger function of LSECs depends on a complete process of endocytosis and clearance [39]. The scavenger capacity of LSECs is compromised by multiple factors, including aging, exposure to drugs and toxins, and imbalance of transcription factors.

### 3.1. Aging

Aging significantly impairs the endocytic and clearing capacities of LSECs [79]. LSECs subjected to hypoxic injury may transition into a senescence-associated secretory phenotype (SASP), while ischemic and hypoxic hepatocytes release high mobility group box 1 (HMGB1), a damage-associated molecular pattern (DAMP), which further promotes LSEC senescence and impairs their clearance functions [80]. A study in rats indicates that SR-H1- and SR-H2-mediated endocytic capacity declines in old rats [39]. These alterations lead to reduced uptake of lipoproteins, potentially resulting in hyperlipidemia. Moreover, age-related pseudocapillarization contributes to a decrease in endocytotic activity in LSECs, which diminishes the clearance of macromolecules such as antibodies and collagen degradation products [79]. Furthermore, it is found that the endocytic ability of aging LSECs to internalize formaldehyde-albumin decreases, a change that may be associated with endothelial layer thickening and a slowing of material transport [79]. Notably, this decline in endocytic activity is likely to contribute to a reduction in the clearance of acetaminophen [81].

### 3.2. Drugs and Toxins

LSECs are highly vulnerable to oxidative stress because of limited antioxidant capacity [82]. A variety of drugs and toxins can cause oxidative stress damage, further compromising the scavenger function of LSECs. It is indicated that the toxic effects of acetaminophen lead to decreased expression levels of LYVE-1 and FcγRIIb in LSECs, thereby impairing LSEC scavenger function [82]. Both acute and chronic alcohol exposure impair the scavenger function of LSECs [83]. Specifically, the administration of alcohol reduces the expression of scavenger receptors on the surface of LSECs and significantly decreases the rate of ligand internalization and degradation [84]. Moreover, hydrogen peroxide impairs the endocytic and clearing capacities of LSECs by generating ROS, while toxic lipid oxLDL can induce an increased pathological expression of LOX-1, with a reduction in both the diameter and porosity of their fenestrae [85]. Disrupting clathrin-mediated endocytosis in LSECs can also compromise their scavenger function. It is revealed that dynamin inhibitors, dynasore and myristyl trimethyl ammonium bromides, suppress LSEC endocytic function and inhibit the clearance of cytomegalovirus [50]. In addition, modified proteins in the body, such as AGEs, can also impair the scavenging function of LSECs. AGEs are products generated through irreversible non-enzymatic reactions between proteins and glucose or reactive dicarbonyls, with the ability to promote oxidative stress, inflammation, and apoptosis, thereby contributing to the development of MASLD, ALD, and liver fibrosis [86]. Notably, AGEs can reduce the number of scavenger receptors in LSECs, decrease cellular endocytic activity, and interfere with the transport of internal vesicles within the cells [87].

### 3.3. Transcription Factors

The scavenger function of LSECs depends on the quantity and function of related receptors on their cell membrane surface [39,88]. Recent studies have demonstrated that transcription factors not only maintain the specific phenotype of LSECs but also regulate the expression of their scavenger receptors [89]. For instance, the overexpression of transcription factor purine-rich box 1 (PU.1) can enhance the expression of scavenger receptors including FcγRIIb, MR and LYVE-1, increasing the endocytic capacity of LSECs [90]. Another transcription factor, runt-related transcription factor 3 (RUNX3), is involved in various developmental processes and lineage determination [91]. Its loss leads to decreased SR-H1/2 expression and thereby exacerbates liver disease [92]. Furthermore, GATA binding protein 4 (GATA4) serves as a master regulator of LSEC differentiation, maintaining both the unique cellular phenotype and endocytic performance [89]. Meanwhile, transcription factor musculoaponeurotic fibrosarcoma (MAF) and meis homeobox 2 (MEIS2) can work synergistically with GATA4, and these three transcription factors act a synergistic effect through their coordinated expression, promoting the expression of multiple scavenger-related receptors such as L-SIGN and MR [91]. Additionally, recent studies have shown that yes-associated protein 1 (YAP1) regulates GATA6 transcription, which in turn controls SR-H expression [93]. Its knockdown enhances GATA6 expression, consequently restoring SR-H expression and attenuating cholestatic liver injury in mice.

## 4. Dysregulated Expression of LSEC Scavenger Receptors Contributes to Liver Disease

Scavenger receptors on LSECs are essential for hepatic clearance and immune regulation. Their dysfunction can impair these processes, thereby driving the progression of liver conditions like MASLD, ALD, liver fibrosis and HCC [4,5,94]. They also mediate the hepatotoxicity of antibody-based drugs [6].

### 4.1. Metabolic Dysfunction-Associated Steatotic Liver Disease

MASLD is now the most prevalent chronic liver disease worldwide, affecting more than a third of the adult population [95]. Projections indicate that by 2040, the prevalence of MASLD among adults may exceed 55% [95]. Although numerous mechanisms have been identified as significant contributors to the development and advancement of MASLD, disturbed hepatic lipid metabolism remains a major driver of this disease [96,97]. Importantly, LSECs show dysregulated scavenger function in MASLD [4]. This alteration of LSECs is closely linked to increased inflammation, lipid accumulation and fibrogenesis in liver, suggesting that dysregulated scavenger function is an early and pivotal event in MASLD pathogenesis [98,99].

**Figure 2:**
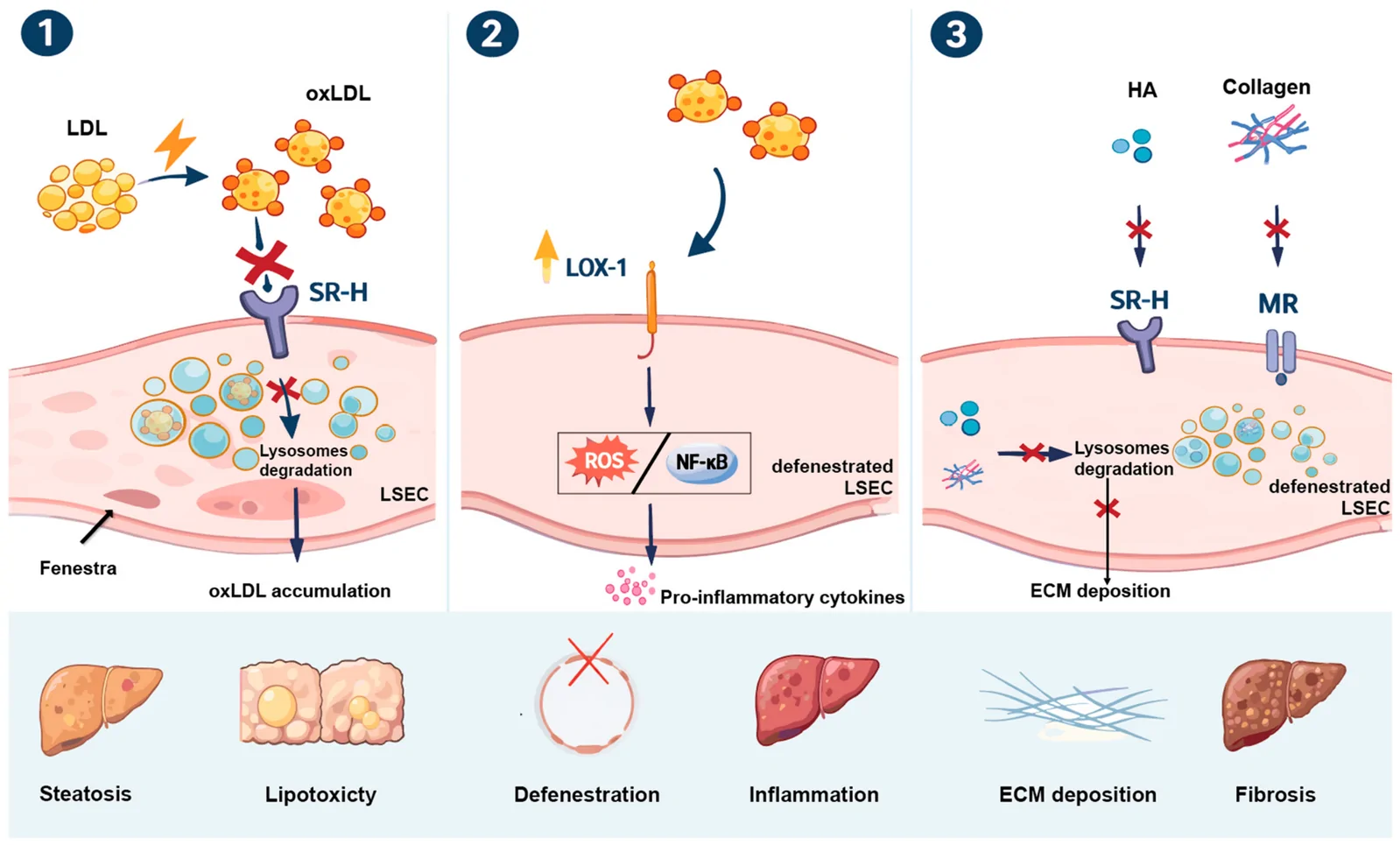
Mechanism of the development of MASLD mediated by dysregulated LSEC scavenger receptors. (**1**) Excess LDL is oxidized to oxLDL, and SR-H dysfunction impairs its lysosomal degradation in LSECs, thereby causing the accumulation of hepatic lipotoxicity. (**2**) Accumulated oxLDL upregulates LOX-1 expression, thereby promoting ROS generation and activating the NF-κB signaling pathway, ultimately leading to the defenestration of LSECs and promoting hepatic inflammatory responses. (**3**) SR-H dysfunction leads to the accumulation of HA, whereas MR dysfunction promotes the deposition of collagen, and these extracellular matrix changes alter the liver microenvironment and contribute to liver fibrosis.

As shown in Figure 2, oxLDL accumulation is an important contributor to the development of MASLD [100]. The dysregulated scavenging function of LSECs significantly reduces their ability to take up and clear circulating oxLDL, leading to abnormal hepatic accumulation of oxLDL and resulting in hepatocellular toxicity [33,100]. Notably, oxLDL can also exert direct toxicity on LSECs through LOX-1 receptor mediation [24]. Specifically, oxLDL significantly upregulates LOX-1 expression in a dose- and time-dependent manner through ROS/NF-κB signaling pathway [101]. This alteration triggers oxidative stress and inflammatory responses, downregulates endothelial nitric oxide synthase (eNOS), and upregulates endothelin-1 (ET-1) and caveolin-1, thereby leading to LSEC defenestration and promoting the progression of MASLD [24,102].

SR-H family comprises critical mediators of hepatic lipoprotein metabolism and cholesterol homeostasis [103]. As the primary scavenger receptors for oxLDL clearance, the two SR-H subfamily members exhibit distinct oxidation-dependent selectivities [33]. Specifically, the SR-H1 maintains consistently high oxLDL uptake rates independent of oxidation level, whereas SR-H2 functions primarily as a receptor for heavily oxLDL. In addition, transcriptomic analyses show that the loss of SR-H in mice triggers alterations in signaling pathways of LSECs, characterized by increased cell adhesion molecules and decreased potential clearance receptors [88]. It reflects the enhancement of local inflammatory responses in the liver and the disruption of the material clearance function of LSECs. Animal experiments show that the knockdown of SR-H achieved by inhibiting the GATA6 transcription factor in LSECs can induce dysregulated lipid metabolism and inflammation, which further exacerbates methionine and choline deficient diet (MCD)-induced MASLD in mice [104].

FcγRIIb mediates the clearance of immune complexes and plays an important role in the regulation of inflammation [39]. A study conducted on patients with MASLD shows that the expression level of FcγRIIb decreases as the disease progresses, and this has also been verified in animal models [98]. Specifically, it is proved that the downregulation of FcγRIIb is associated with the disorder of lipid metabolism and enhanced inflammation response. However, the expression of FcγRIIb did not significantly differ across pathological MASLD grades and the expression of various scavenger receptors is also not coordinated in MASLD [10,98].

### 4.2. Alcoholic Liver Disease

ALD accounts for a substantial proportion of liver disease globally, and is responsible for nearly half of all cirrhosis-related deaths and a significant share of disability-adjusted life years lost [105]. Recent studies have shown that the onset and progression of ALD are closely associated with the dysregulated scavenging function of LSECs.

**Figure 3:**
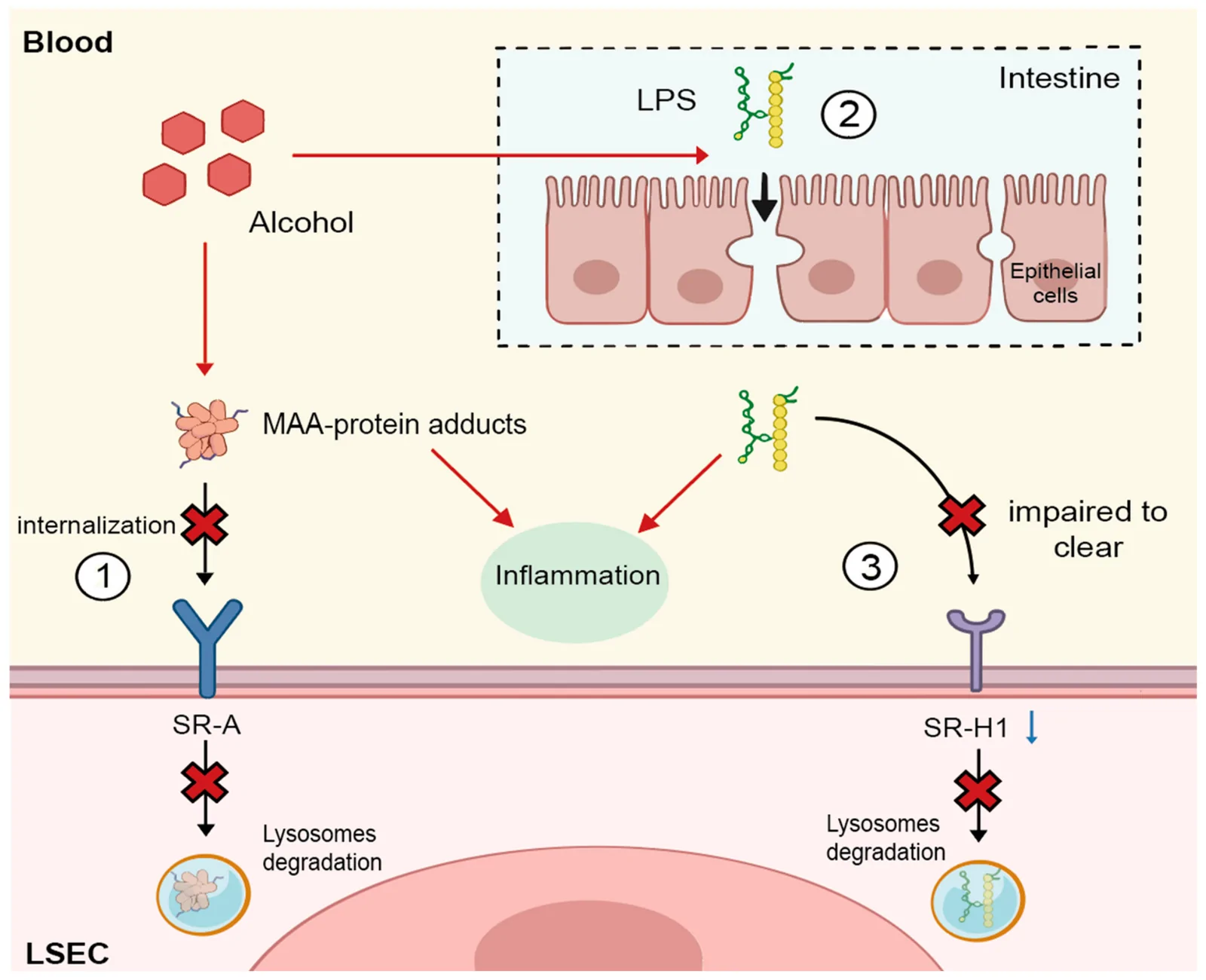
Mechanism of the development of ALD mediated by dysregulated LSEC scavenger receptors. (**1**) Alcohol induces liver inflammation by promoting the production of MAA protein adducts and inhibiting their internalization by SR-A. (**2**) Alcohol damages the intestinal barrier, causing gut-derived LPS to migrate to the portal vein and is subsequently delivered to the liver, triggering an inflammatory response and thereby promoting the progression of ALD. (**3**) The downregulation of SR-H1 in LSECs leads to LPS clearance impairment, ultimately promoting ALD progression.

In the early stages of alcohol-induced liver injury, alcohol initially impairs the scavenger function of LSECs [106]. For instance, alcohol can affect the internalization of HA, a critical biomarker of LSEC clearance function. This dysfunction precedes elevations in alanine aminotransferase (ALT) levels and morphological injury to hepatocytes [83]. Then, the accumulation of harmful substances ensues, which plays a critical role in the pathogenesis of ALD [107]. MAA-SA, a highly reactive adduct generated during alcohol metabolism, is specifically recognized and cleared by LSECs under physiological conditions [5,108]. However, when this clearance pathway is compromised, the accumulated malondialdehyde–acetaldehyde–serum albumin adduct (MAA-SA) acts as a damage amplifier in the onset and progression of ALD by stimulating hepatic inflammatory responses [108]. Significantly, as shown in Figure 3, chronic alcohol consumption has been shown to accelerate ALD progression by suppressing SR-A-mediated clearance of MAA-SA [108]. It suggests that alcohol primarily causes the accumulation of MAA-SA by inhibiting its internalization process, rather than directly suppressing its degradation [5].

Beyond impairing the clearance of toxic protein adducts, alcohol also disrupts the intestinal barrier, permitting the translocation of gut-derived LPS into the portal circulation and subsequent delivery to the liver, thereby promoting the progression of ALD [109]. Under physiological conditions, LSECs serve as a critical second line of defense against circulating LPS through receptor-mediated scavenging. However, when LSEC scavenging function is compromised by chronic alcohol exposure, the detrimental effects of LPS on hepatic tissue are markedly exacerbated [34]. For instance, the SR-H1 receptor expressed on LSECs has been demonstrated to mediate the clearance of LPS and regulate hepatic inflammatory responses [34]. Accordingly, the impairment of this receptor contributes significantly to the progression of ALD [110]. Thioredoxin-interacting protein (TXNIP), a stress-response protein, is instrumental in cellular growth and differentiation [111]. Evidence demonstrates that the loss of TXNIP suppresses the eNOS signaling pathway, resulting in a significant reduction in the expression of the scavenger receptor SR-H in LSECs, accompanied by capillarization of these cells. These pathological alterations subsequently exacerbate ALD [112].

### 4.3. Liver Fibrosis

Liver fibrosis is a common pathological manifestation of various chronic liver diseases, distinguished by the excessive accumulation of the extracellular matrix [2]. Epidemiological research shows that the disease burden of liver fibrosis is significant, and is particularly high among individuals with diabetes, obesity, and fatty liver disease, and the prevalence of advanced fibrosis and cirrhosis is increasing [113].

SR-H1 and SR-H2 are the most important multi-ligand scavenger receptors in LSECs, responsible for clearing hyaluronic acid, procollagen peptide, oxidized lipoprotein, and various damage-associated macromolecules, thereby directly linking their dysfunction to fibrotic matrix accumulation [23,32,33,34,35]. Transcriptomics reveal that single or double deletion of SR-H1 and SR-H2 remodels the function of LSECs, upregulating inflammation and fibrosis-related pathways [88]. Moreover, evidence from animal experiments also shows that defects in SR-H1 and SR-H2 can exacerbate thioacetamide-induced liver fibrosis or cause mild perisinusoidal fibrosis in the liver of normal mice [92,114].

FcγRIIb also plays an important role in liver fibrosis. Clinical research shows that the downregulated expression of FcγRIIb leads to an elevation of liver fibrosis markers including type IV collagen and hyaluronan, and it has also been verified in animal models [98]. Additionally, evidence suggests that FcγRIIb-mediated endocytosis of soluble immune complexes is significantly reduced in Glmp (gt/gt) fibrotic livers, which promotes the amplification of inflammation and exacerbates the development of liver fibrosis [4]. Moreover, other receptors such as LSECtin, LYVE-1, and MR are also downregulated in liver fibrosis [77,88,92].

### 4.4. Hepatocellular Carcinoma

HCC is the most common type of liver cancer, with a high mortality rate and low five-year survival rate, placing a substantial burden on healthcare systems worldwide [115]. The occurrence and progression of HCC are influenced by various etiological risk factors, including HBV and HCV infections, MASLD, ALD, and tobacco use [115]. Recent studies have shown that LSEC scavenger receptors play a crucial role in the immune evasion and metastasis of HCC tumor cells.

LSECs maintain immune homeostasis within the liver through scavenger receptors, by modulating the balance between immune response and immune tolerance [78]. Under pathological conditions, however, LSEC dysfunction can lead to T cell exhaustion, accompanied by an upregulation of immunosuppressive molecules [74]. Furthermore, the impaired scavenger function reduces the ability of LSECs to clear harmful substances such as immune complexes and toxic lipids [4]. This reduction not only promotes chronic inflammation and fibrosis but also establishes a permissive environment for the development of HCC [4]. Consequently, the dysregulation of scavenger function in LSECs exacerbates liver injury and undermines anti-tumor immunity [77], thereby promoting the progression of HCC [74].

**Figure 4:**
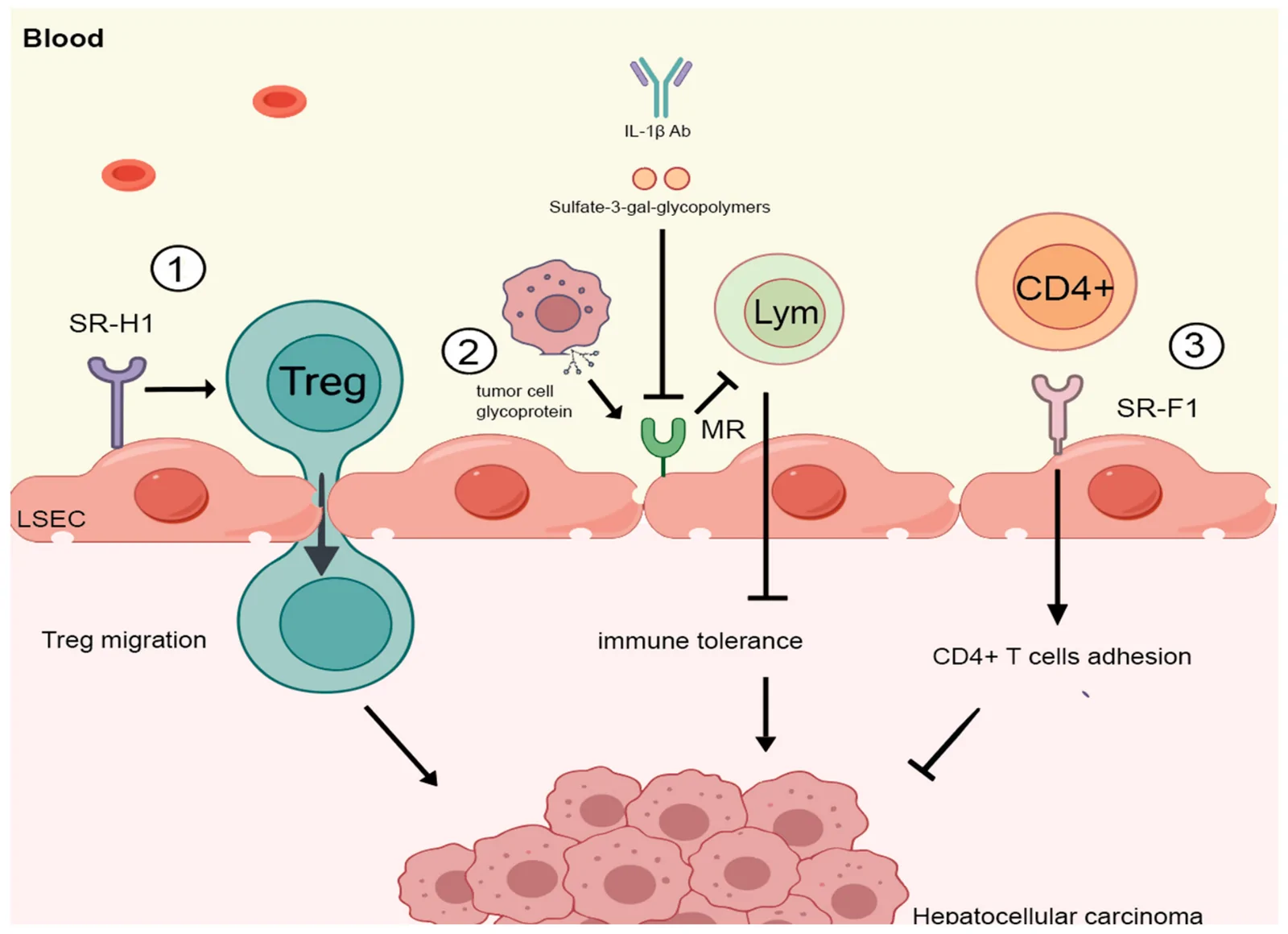
Dysfunction of scavenger receptor in LSECs promotes the progression of HCC. (**1**) SR-H1 promotes Treg migration, mediates tumor cell immune escape, and subsequently promotes HCC progression. (**2**) MR mediates immune tolerance by recognizing tumor glycan antigens and presenting them to lymphocytes, and this effect can be inhibited by IL-1β neutralizing antibodies and sulfate-3-gal-glycopolymers. (**3**) The downregulation of SR-F1 during HCC progression compromises the recruitment of CD4^+^ effector T cells mediated by LSECs.

Specifically, as shown in Figure 4, LSEC scavenger receptors primarily regulate the development of HCC by recruiting various types of immune cells. For instance, SR-H1 in LSECs enhances the migration of regulatory T cells across these endothelial cells, thereby contributing to tumor cell immune evasion [94]. Clinical evidence also indicates that there is a loss of expression of SR-H1 and SR-H2 in the surrounding tissues of liver cancer lesions, and this loss is correlated with increased survival time [94]. Similarly, the increased expression of SR-F1 in chronic liver inflammation can promote the adhesion of CD4^+^ T cells and exacerbate the inflammatory response [94]. However, in HCC, the expression of SR-F1 is significantly reduced in HCC tumor tissues, and its low expression is associated with impaired recruitment of CD4^+^ effector T cells, which positively correlates with tumor aggressiveness and poor prognosis [31]. In addition, the augmented function of MR in LSECs plays a pivotal role in tumor immune evasion by mediating immune tolerance [116]. It is indicated that sulfate-3-gal-glycopolymer possesses the capacity to inhibit MR function, thereby presenting a promising therapeutic strategy for oncological treatment [77]. In addition, research demonstrates that IL-1β neutralizing antibodies can effectively prevent the upregulation of MR, thereby attenuating T lymphocyte immunosuppression mediated by tumor-activated LSECs. This mechanism is vital in inhibiting colorectal cancer liver metastasis [116,117].

### 4.5. Antibody-Based Drug-Related Liver Injury

In recent years, antibody-based drugs have become widely used for treating conditions like cancer, metabolic disorder and autoimmune disease [118]. While DILI is frequently associated with antibiotics, emerging evidence highlights that some antibody-based drugs, especially immune checkpoint inhibitors, can also cause liver injury [119]. This occurs through indirect mechanisms, primarily due to excessive clearance of circulating immune complexes, which leads to tissue damage and inflammation [119,120].

The scavenger function of LSECs is closely related to liver diseases induced by antibody-based drugs, particularly through the FcγRIIb-mediated antibody clearance [119]. Expressed on LSECs, FcγRIIb serves as a cross-linking scaffold that excessively activates CD8^+^ T cells, thereby increasing the hepatotoxicity associated with humanized antibodies designed to enhance antitumor immune responses [6]. Studies have shown that urelumab, a potent CD137 agonist, necessitates the involvement of FcγRIIb for its hepatotoxic effects [6]. Furthermore, specific antibody-based drugs can elicit hepatic inflammatory responses, accompanied by the accumulation of immune deposits within hepatic sinusoids, and may even manifest as sinusoidal obstruction syndrome [121,122].

## 5. Pharmacological Approaches to Restore LSEC Scavenger Function

Although the scavenger function of LSECs plays an important role in the onset and progression of liver diseases, pharmacological research on regulating the expression and function of scavenger receptors in LSECs remains limited. Recent studies have shown that certain drugs can restore the expression and function of scavenger receptors on LSECs by alleviating oxidative stress, inhibiting angiogenesis, and modulating immune and inflammatory responses [123,124,125]. The investigated agents, LSEC-associated receptors, and evidence levels are summarized in Table 2.

### 5.1. Drugs to Alleviate Oxidative Stress Damage

Oxidative stress arises predominantly from mitochondrial overproduction of ROS [126]. This pathological condition impairs scavenger receptor-mediated endocytosis and lysosomal degradation functions of LSECs, leading to the accumulation of harmful substances in blood [85]. Evidence suggests that targeting ROS reduction represents a potential strategy to protect the scavenger function of LSECs from oxidative injury and subsequently alleviate liver disease.

N-acetylcysteine (NAC) is a commonly employed antioxidant. Although its direct ROS scavenging capacity is limited under physiological conditions due to minimal thiol group dissociation, NAC confers substantial antioxidant benefits through the promotion of glutathione (GSH) replenishment [123]. In isolated rat LSECs, NAC pretreatment limited subsequent hydrogen-peroxide injury, but it did not reverse established damage, and intracellular degradation was more affected than receptor-mediated uptake [85]. In addition, neither receptor specific target engagement nor LSEC scavenging capacity has been evaluated as a clinical endpoint [123].

Melatonin, apart from regulating circadian rhythms, has also been found to possess antioxidant and anti-inflammatory properties [127]. Recent research indicates that melatonin can restore the expression of LYVE-1 in LSECs and improve hepatic microcirculation, which is of significant importance for the development of chronic hepatic diseases [128].

Acteoside, a hydrophilic phenylethanoid glycoside structurally composed of caffeic acid, hydroxytyrosol, glucose, and rhamnose, exhibits potent anti-inflammatory and antioxidant pharmacological effects [129]. Research indicates that acteoside blocks the binding of SASP factors, specifically high mobility group protein B1, to LSECs. By restoring the endocytic capacity and preserving the physiological phenotype of LSECs, acteoside demonstrates therapeutic potential in the management of aging-related liver diseases [80].

### 5.2. Drugs to Inhibit Angiogenesis

As important pro-angiogenic factors, vascular endothelial growth factor (VEGF) and platelet derived growth factor (PDGF) are indispensable for preserving the phenotype of LSECs [130]. However, dysregulated expression of these factors can trigger pathological angiogenesis and drive LSECs toward pathological capillarization, with impaired scavenger function [124,131]. Recent studies suggest that certain drugs can inhibit the VEGF or PDGF signaling pathway, thereby restoring the scavenger function of LSECs and suppressing the progression of liver disease.

YC-1, a small molecule initially developed as an activator of soluble guanylate cyclase (sGC) [132], has gained attention for its broad pharmacological activities, including antiplatelet, vasodilatory, anti-inflammatory, and antitumor effects [133]. Animal experiments indicate that YC-1 upregulates the expression of LYVE-1 in LSECs by inhibiting the expression of PDGF-BB, accompanied by amelioration of liver fibrosis [124].

Dark tea is a type of post-fermented tea [134]. During the fermentation process of dark tea, bioactive components with unique pharmacological activities are formed, including tea polyphenols, tea polysaccharides, thearubigins and related tea pigments, flavonoids, alkaloids, and peptide substances [134]. These components confer a variety of pharmacological effects on dark tea, such as anti-inflammatory activity, prevention of cardiovascular diseases, and liver protection [135]. Research shows that dark tea extract significantly inhibits pathological angiogenesis in thioacetamide-induced liver fibrosis in mice by downregulating VEGF-A. This effect can restore the expression of LYVE-1 in LSECs and is of great significance for the treatment of liver fibrosis [131].

### 5.3. Drugs to Modulate Inflammatory Responses

Tissue injury triggers inflammatory responses through pro-inflammatory mediators like tumor necrosis factor-α (TNF-α) and IL-1β released by immune cells and damaged epithelial or endothelial cells [136]. These mediators contribute to the upregulation of LOX-1 expression in LSECs, which subsequently activates NF-κB signaling pathways and further induces the expression of inflammatory cytokines and cell adhesion molecules, forming a positive feedback loop of inflammation and playing an important role in MASLD [137,138]. Therefore, directly inhibiting the expression or function of LOX-1 is of great significance for alleviating hepatic inflammation and inhibiting the progression of liver disease [139].

Amygdalin is a cyanide-releasing compound found mainly in fruit kernels [140]. Recent studies have clarified its important roles in anti-inflammatory, anti-tumor and anti-fibrotic activities [141]. Notably, study has indicated that it ameliorates MASLD by mitigating inflammation, specifically through the suppression of IL-1β and arachidonic acid production, and by upregulating SR-H2 to restore lipid homeostasis in LSECs [125].

Si-Wu-Tang (SWT), a traditional classic formula comprising Chuanxiong Rhizoma, Paeoniae Radix Alba, Radix Angelicae Sinensis and Rehmanniae Radix Praeparata, is widely employed in clinical practice for the management of gynecological dysfunctions [142]. Recent pharmacological investigations have extended the clinical scope of SWT, revealing its significant efficacy in treating hepatobiliary pathologies [143]. Research demonstrates that the primary pharmacological action of SWT is to downregulate the expression of LOX-1, thereby attenuating LOX-1–mediated endothelial injury and inflammatory responses and consequently inhibiting the progression of hepatic fibrosis [144].

In addition, suppressing IL-1 expression can regulate MR expression in LSECs [145]. Celecoxib, a selective cyclooxygenase-2 (COX-2) inhibitor, is widely recognized for its potent anti-inflammatory, antipyretic, and analgesic properties [146]. Notably, recent studies have shown its capacity to inhibit metastatic progression in various malignancies like colorectal, breast, and prostate tumors [147,148]. Mechanistically, celecoxib disrupts the crosstalk between tumor cells and LSECs by inhibiting COX-2 activity [148]. This inhibition suppresses the secretion of IL-1 from LSECs and prevents the upregulation of the MR, thereby thwarting tumor immune evasion and effectively inhibiting hepatic metastasis [117].

### 5.4. Clinical Translation and Limitations

The evidence supporting these approaches is largely derived from in vitro and animal studies, and none of the strategies discussed above has been clinically validated specifically for restoring LSEC scavenger function. The feasibility analysis of the drug translation will be carried out as follow. Firstly, NAC, melatonin and celecoxib are clinically available for other indications, but their effects on LSEC endocytosis or scavenger receptor expression have not been elucidated in patients [85,128,148]. Their established clinical availability and safety facilitate translational research [149,150,151]. Future studies should establish efficacy in relevant liver diseases and determine whether these agents improve LSEC endocytosis or scavenger receptor expression. Secondly, dark tea and SWT are orally administered preparations, represented by a fermented beverage and a traditional classical formula, respectively [134,142]. Their safety profiles are relatively manageable, supporting their potential for clinical translation [152]. Rigorous preclinical studies should assess efficacy, pharmacokinetics, and safety before clinical testing. Thirdly, the clinical translation of YC-1 and amygdalin is limited by their considerable toxicity [141,153]. As for acteoside, it is unstable and has low oral bioavailability, limiting its clinical application [129]. Of note, the strong endocytic capacity of LSECs and their sensitivity to particle size and surface properties also provide a promising rationale for the development of liver sinusoid-directed delivery systems [154]. Targeting LSEC scavenger pathways may not only improve intrahepatic drug accumulation and therapeutic efficacy, but also reduce extrahepatic exposure and systemic toxicity [155]. For these drugs, future research should focus on two major strategies, namely structural optimization and liver-targeted drug delivery systems, to improve bioavailability and reduce systemic toxicity [132].

## 6. Discussion

LSECs are essential regulators of hepatic homeostasis, and their scavenger function represents a fundamental mechanism through which the liver maintains metabolic balance, removes circulating waste and potentially harmful substances, and coordinates local immune tolerance and inflammatory responses. As summarized in this review, the broad repertoire of scavenger receptors expressed on LSECs enables these cells to clear a wide range of endogenous and exogenous ligands, including modified lipoproteins, immune complexes, extracellular matrix components, pathogen-associated molecules, and drug-related substances. Through these activities, LSECs contribute to the preservation of sinusoidal integrity and the maintenance of a stable hepatic microenvironment.

Increasing evidence indicates that impairment of LSEC scavenger function is closely involved in the development and progression of multiple liver diseases. Aging, oxidative stress, toxins, transcription factors dysregulation and the accumulation of modified proteins can compromise receptor-mediated endocytosis or degradative capacity in LSECs. These alterations promote the intrahepatic accumulation of lipotoxic, pro-inflammatory, and pro-fibrotic mediators, thereby facilitating the progression of MASLD, ALD and liver fibrosis. Furthermore, impaired immune regulation resulting from dysregulated LSEC scavenger function is also significantly associated with the development of HCC. In addition, the endocytic function mediated by LSEC FcγRIIb can alter the distribution of antibody-based drugs and contribute to treatment-related liver injury. However, although several scavenger receptors are dysregulated in liver diseases, their changes do not appear to follow a consistent pattern [4,18]. For instance, a study shows that FcγRIIb expression is reported to be decreased in a mouse model of liver fibrosis, whereas SR-H and MR expression shows no significant changes [4]. In contrast, other studies find that MR expression is reduced during liver fibrosis and knockout of SR-H can directly cause liver fibrosis [92,156]. These discrepancies may be due to differences between mouse and human samples, as well as variations in the methods used to induce liver fibrosis.

Emerging pharmacological approaches targeting oxidative stress, angiogenesis, and inflammation show promise in restoring LSEC scavenger function. However, the field remains at an early stage. Most studies are still descriptive or preclinical, and many mechanistic questions remain unresolved, including how individual scavenger receptors coordinate ligand selectivity, intracellular trafficking, receptor crosstalk, and communication with hepatocytes, KCs, HSCs, and infiltrating immune cells under different pathological conditions. Moreover, the disease specific and stage dependent roles of distinct scavenger receptors have not yet been fully defined, which limits the precision of current therapeutic strategies.

The evidence supporting LSEC scavenger receptors as potential biomarkers remains dominated by preclinical studies, while clinical observations in MASLD, ALD, liver fibrosis, and HCC also indicate their potential clinical relevance [124,156]. In the animal studies summarized above, LYVE-1, SR-H, and LOX-1 were dysregulated under disease conditions but were restored following pharmacological intervention, in parallel with improvements in liver pathology [124,125,128,131,144]. These findings suggest that scavenger receptor profiles may serve as responsive indicators of LSEC functional status. Clinical findings further support this possibility. In patients with ALD, elevated plasma levels of soluble MR are associated with greater disease severity [157]. In MASLD, analysis of liver samples from 26 patients showed that LSEC FcγRIIb expression was inversely correlated with measures of hepatic lipid accumulation and fibrosis, although it did not differ significantly across overall histopathological grades [98]. Similarly, in liver fibrosis, reduced MR expression in LSECs is closely associated with fibrosis severity [156]. As for HCC, higher endothelial SR-F1 expression is associated with less aggressive disease and improved survival [31,158]. Despite close links between dysregulated LSEC scavenger receptors and liver diseases, their current diagnostic or prognostic utility still remains difficult to establish, because these receptors have limited cellular and disease specificity. For instance, circulating MR signals can correlate with or arise from monocyte/macrophage source, and the expression of this circulating MR is more relevant to inflammation, making it difficult to differentiate [156,157]. Therefore, scavenger receptors may not serve as reliable independent biomarkers. However, they can be used as diagnostic or prognostic biomarkers in combination with other biomarkers of liver diseases, such as hyaluronic acid and ox-LDL.

The application of new technologies such as single-cell sequencing on LSECs is gradually expanding. Human single-cell RNA sequencing (scRNA-seq) identified LSEC-enriched expression of FcγRIIb, LYVE-1, stabilin1/2, and LSECtin, with FcγRIIb protein expression being particularly prominent in midzonal and pericentral sinusoids [159]. In cirrhosis, scRNA-seq showed a loss of endocytic receptor programs in pericentral mouse LSECs [160], while human scRNA-seq with tissue mapping showed depletion of CD34-negative LSIGN-positive LSECs and expansion of PLVAP-positive/ACKR1-positive scar-associated endothelia [161]. Additionally, through spatial localization, a study suggests that the MAF gene is predominantly expressed in midzonal LSECs and regulates their normal physiological functions [162]. This finding may explain the development of midzonal fibrosis observed in MAF-knockout mice. Nevertheless, direct spatial transcriptomic analyses focused on LSECs in liver disease remain lacking. Given the heterogeneous distribution of LSEC subpopulations along the liver sinusoids [160], integrating scRNA-seq with spatial transcriptomics could map disease-associated LSEC states along the porto–central axis, define where scavenger, inflammatory, angiocrine, and capillarization programs emerge, and identify spatially restricted interactions with hepatocytes, KCs, and HSCs. Such analyses could distinguish true cell-state changes from altered zonal composition, link molecular programs to fibrotic niches, and reveal zone-specific biomarkers or therapeutic targets.

Therefore, future research should focus on clarifying the receptor-specific mechanisms of LSEC scavenging in different liver diseases, validating key findings in human tissues and clinically relevant models, identifying diagnostic or prognostic biomarkers of liver diseases, and conducting spatial transcriptome analysis of LSECs in liver disease. A deeper understanding of LSEC scavenger biology may thus open new avenues for both mechanistic insight and translational intervention in liver disease.

## Figures and Tables

**Table 1:** Structural features, representative ligands and principal functions of LSEC scavenger receptors.

Receptor(s)	Classification	Representative Ligands or Cargo	Principal Role in LSECs	Refs.
SR-A	type II transmembrane	Modified LDL, LPS, β-amyloid fibrils, AGEs and MAA-SA	Bind anionic cargo	[18,19]
SR-B1 and SR-B2	type II transmembrane	SR-B1: HDL, VLDL, fat-soluble vitamins and HCV; SR-B2: oxLDL, anionic phospholipids and collagen	Uptake lipoprotein and cholesteryl-ester	[19,20,21,22]
SR-E1 (LOX1)	type II transmembrane	oxLDL	Clear inducible oxLDL; low basal expression but marked upregulation under pathological conditions	[19,23,24]
SR-E3 (MR)	type I transmembrane	Terminal mannose, fucose and N-acetylglucosamine; FSA; pathogen glycoproteins; tissue plasminogen activator, procollagen, peroxidase, hemoglobin and lysosomal enzymes	Clear glycoconjugates, pathogens and endogenous proteins	[25,26,27,28]
SR-F1	type I transmembrane	Modified LDL, heat-shock proteins, apoptotic cells and microbial antigens	Link to intracellular signaling and immune tolerance	[29,30,31]
SR-H1 and SR-H2 (stabilin-1/2)	type I transmembrane	Shared: phosphorothioate oligonucleotides, HOA and phosphatidylserine; SR-H1: oxLDL and LPS; SR-H2: heparin, HA, chondroitin sulfate, acetylated LDL	Major LSEC scavenger receptors; uptake and degrade anionic and oxidized macromolecules	[23,32,33,34,35]
SR-L (LRP1)	type I transmembrane	α2-macroglobulin, receptor-associated protein, β-amyloid fibrils, LDL and VLDL	Maintain lipid homeostasis in cooperation with other scavenger receptors	[23,36,37,38]
FcγRIIb	type I transmembrane	IgG and IgG-containing immune complexes	Degrade immune complexes	[39,40]
LSECtin and L-SIGN	type II transmembrane	LSECtin: mannose-, N-acetylglucosamine- and fucose-bearing glycoproteins; L-SIGN: viral glycoproteins and vWF-factor VIII complex	Recognize glycoprotein and pathogen	[39,41,42,43,44,45,46]
LYVE-1	type I transmembrane	HA	Marker of LSEC differentiation; Clear HA	[47,48,49]

Abbreviations: AGEs, advanced glycation end products; FSA, formaldehyde-treated serum albumin; HA, hyaluronic acid; HCV, hepatitis C virus; HDL, high-density lipoprotein; HOA, highly oxidized albumin; IgG, immunoglobulin G; LDL, low-density lipoprotein; LOX-1, lectin-like oxidized low-density lipoprotein receptor-1; LRP1, low-density lipoprotein receptor-related protein 1; MAA-SA, malondialdehyde-acetaldehyde-serum albumin adduct; MR, mannose receptor; oxLDL, oxidized low-density lipoprotein; VLDL, very-low-density lipoprotein; vWF, von Willebrand factor.

**Table 2:** Therapeutic agents investigated for restoring or modulating LSEC scavenger function in liver disease.

Drug	LSEC Receptor	Associated Liver Disease	Level of Evidence	Proposed Mechanism of Action
NAC	endocytic capacity	MASLD	In vitro and animal	Alleviate oxidative injury
Melatonin	LYVE-1	Chronic liver disease	Animal	Alleviate oxidative injury
Acteoside	endocytic capacity	Hepatic ischemia- reperfusion injury	In vitro and animal	Alleviate oxidative injury and inhibit inflammatory response
YC-1	LYVE-1	Liver fibrosis	In vitro and animal	Inhibit pathological angiogenesis
Dark tea	LYVE-1	Liver fibrosis	In vitro and animal	Inhibit pathological angiogenesis
Amygdalin	SR-H2	MASLD	In vitro and animal	Reduce prostaglandin E2, suppress Ca^2+^ overload and inflammatory response
SWT	LOX-1	Liver fibrosis	In vitro and animal	Inhibit pathological angiogenesis and inflammatory response
Celecoxib	MR	Colorectal cancer liver metastasis	In vitro and animal	Inhibit COX-2-dependent IL-1 signaling, limit MR upregulation and suppress immune tolerance

## Data Availability

No new data were created or analyzed in this study. Data sharing is not applicable to this article.
